# Towards transforming teaching and learning in higher education: interrogating poverty through a decolonial perspective

**DOI:** 10.1007/s41297-023-00188-w

**Published:** 2023-05-10

**Authors:** Otilia Chiramba, Shireen Motala

**Affiliations:** Faculty of Education, University of Johannesburg, Johannesburg, South Africa

**Keywords:** Decoloniality, Epistemic disruption, Material poverty, Teaching and learning

## Abstract

This paper aims to address three questions relating to the imperatives of knowledge transformation, the impact of poverty broadly and material poverty in particular, and questions of pedagogy or approaches to delivering knowledge in higher education institutions. Transformation of teaching and learning in higher education is at a crossroads in South Africa. A major driver of this is the recently emerged student-led protests on decolonisation. At these crossroads are a number of factors with the potential to facilitate or inhibit the decolonisation process. First is a conceptual paralysis regarding understanding of the idea in the country’s higher education. Second are competing ideological imperatives which confound universities’ strategic choices. Third is the fact that the academy has a high proportion of staff who themselves are products of the system they hope to transform. The paper utilises conceptual and theoretical arguments to explore the state of teaching and learning in South Africa and to learn how the protests have enhanced or/and diminished educational gains among students who are poverty stricken and facing deprivation. The paper argues for a theory of decoloniality to shape thinking and practices which enable higher education institutions to transform teaching and learning and specially to drive the type of transformation which enhances poor students’ epistemological access. The paper recommends investment in further research to explore the impact of material poverty in higher education and how the transformation of knowledge and pedagogy could be better understood for the majority of students who live with poverty in higher education.

## Introduction

In post-apartheid democratic South Africa, decolonisation has mainly focused on transforming education and on higher education specifically. The argument that knowledge, and the standards that determine its validity, has been disproportionately informed by Western systems of thought and ways of thinking is not new in the context of decolonisation. Gwaravanda (2019) argues that this kind of knowledge has implications for the African way of knowing.

Decolonisation is not a new concept and is commonly used to mean transition from and resistance to colonialism. In education, decolonisation involves tackling and dismantling “the epistemic violence and hegemony of Eurocentrism, completely rethink[ing], refram[ing] and reconstruct[ing] the curriculum and plac[ing] South Africa, Southern Africa and Africa at the centre of teaching, learning and research” (Heleta, 2016, p. 3). In education and higher education specifically, we recognise the decolonisation ideology as posing questions around what knowledge is of greatest worth, what content we should be teaching, how education should be delivered to students from different socioeconomic backgrounds and how these students should be assessed. Fiske and Ladd (2004) argue that knowledge transformation is necessary if South Africa is to build a racially equitable society. There is great need to maintain a keen focus on eradicating the hegemony of the Western canon which has tended to reproduce and cement inequalities and epistemological injustices in post-colonial higher education institutions.

Voices for the decolonisation of higher education in formerly colonised spaces, including on the African continent, have been increasing in intensity in recent years (Jansen, 2019; Maringe, 2020; Rensburg et al., 2020). The focus on the politics of knowledge which is central to the edited book by Jansen (2019) is a legitimate and an important one and provides a useful means by which the universities could interrogate the notion of decolonisation especially in relation to the student protests of 2015/2016. These are an important focus of this paper. Jansen’s book has dealt mainly with coloniality of knowledge, driven largely by the neo-liberal argument, which in itself is an important dimension of the capitalist project of the West (Jansen, 2019). However, whilst Jansen’s argument is generative in the sense of identifying strategies that confront neo-liberalism in higher education, it seems to have ignored the context in which higher education operates especially in the post-colonial world. We believe that one of the key aspects of this context is the persistent phenomenon of poverty. We argue that as long as poverty is not interrogated in its various multi-dimensional factors, the project of decolonisation of higher education might simply reproduce the asymmetries of power, knowledge and being which the project of neo-liberalism and capitalism has firmly established in our universities and societies. This paper focuses on the aspect of poverty and its three dimensions of material, economic and epistemological deprivations and their impact on the decolonisation project in higher education (Motala et al., 2021). As Bawa (2020, p. 49) has rightly said, the three dimensions of poverty are among the issues that sparked the 2015/2016 student protests:

Among the many students’ activist voices at the time, one heard a powerfully articulated view that the higher education system was a part of the socio-political infrastructure that produced and maintained a society of deep inequality, grinding poverty, corruption and, ultimately, the erosion of the promises of the Freedom Charter and the struggle for democracy. One has to ask whether it is time to think more specifically about a social justice agenda for higher education: broadening the access and success of students, addressing the decolonisation of its knowledge project and maximising the opportunity for the intellectual, physical, social and emotional development of its students; thereby reconfiguring its relationship with its publics.

Whilst we acknowledge Jansen’s (2019) focus on higher education principles of the politics of knowledge in universities, in our view, he did not place sufficiently robust emphasis on how this knowledge is created and transmitted within higher education through research, teaching and learning amidst the poverty which has a huge impact on the education system as a whole.

This paper focuses on the intersectionality among three factors of poverty and deprivation, epistemologies and knowledge systems and modalities of teaching and learning in higher education to examine how these factors combine to create different modes of discrimination and privilege. The paper also examines the kind of decolonisation advocated by students in the RhodesMustFall (#RMF) and the FeesMustFall (#FMF) protests. These protests were motivated by the economic and material deprivation that the majority of the students were facing. #RMF started at Rhodes University when students wanted the removal of the statue of Rhodes because for them, it was a symbol of perpetual colonialism (Nyamnjoh, 2021). #FMF was the name attached to the subsequent protests among students at universities across South Africa and was provoked by a fee increase of 10.5% at the University of the Witwatersrand in 2015/2016 (Chikane, 2018). The needs for free education and decolonisation of higher education were at the heart of the protest (Chikane, 2018). Mavunga (2019) argues that student protests of this nature are an extension of an unresolved past and persistent poverty.

#FMF and #RMF gave the spark to the extensive and substantive debates around the decolonisation project in South African higher education. Below, we indicate the gaps in literature relating to student-led protest.

Firstly, we lack sufficient knowledge of how issues of poverty and deprivations impact on students’ navigation of and persistence to progress in higher education. Secondly, we are not sufficiently informed about what knowledge is of value and how students may be supported to reach the highest level of participation and engagement in knowledge creation as demanded by the universities. Lastly, we are limited in our knowledge of how to conduct effective teaching and learning among students facing material poverty. The paper thus aims to expand the theoretical and conceptual knowledge base for understanding the impact of disruptions in higher education with a focus on the impact of poverty and its dimensions of material, economic and epistemological deprivations and how they impact on teaching and learning and on students’ knowledge development. The debates raised by the #FMF and #RMF movements question whether the South Africa higher education system has sufficiently transformed in at least three ways and this constitutes the three questions central to this paper:

- What is the impact of poverty and deprivation on teaching and learning in higher education?
- What knowledge transformation is needed to deal more effectively with contextual issues within universities?
- How have different modalities of teaching and learning been incorporated in universities to address challenges for students facing material deprivation?

## Decolonisation within a context of poverty and deprivation

The aforementioned positions are the authors’ constructions based on theoretical and conceptual literature in the field. We argue that the debates on decolonisation are scattered and lacking and consequently only provide a partial answer to the problem. We further argue that, despite the move to a decolonisation agenda as brought by the #FMF movement, the academy has only meaningfully addressed the symbolic dimension of transformation with less focus on how students from low socioeconomic backgrounds can be recognised within the epistemological and ideological dimensions of decolonisation. We theorise the debates and bring them together with the aim of creating a starting point for compiling evidence and adding knowledge to the long-standing debate of decolonisation.

Twenty-one years after the introduction of the decolonisation agenda, students, through protests, demanded that the curriculum be based on Afrocentric principles, making reference to African authors. We view this kind of decolonisation led by student protests as a crisis that has brought many disruptions in the academy. The protests raised questions about epistemes and structures of power and knowledge. Despite the negative impact, they awakened researchers and university leadership to the realisation that the decolonisation project has been slow and that it is necessary to develop more viable solutions.

An extended definition of decolonisation in education involves putting forth the needs of all students and being responsive to the particular needs of each student. Devlin (2013) has argued that when students from low socioeconomic backgrounds succeed at university, all students succeed. However, as argued by one scholar, students from low socioeconomic backgrounds are not well positioned in relation to social and cultural capital that enables them to negotiate the educational strategies that facilitate university (Tranter, 2012). This has also been argued in Bourdieu’s popular theory of social capital and habitus (Bourdieu, 1977). His main argument is that students from low socioeconomic backgrounds lack cultural, linguistic, economic, social and symbolic capital that is highly valued in education. The epistemological and methodological trends in the scholarship of student access and success heavily depend on global theoretical influences which are detached from contextual realities and therefore need de-colonial approaches that reflect social justice as well as accounting for the contextual peculiarities of students’ agency and experience (Cross & Govender, 2021). Thus, the concept of poverty and deprivation plays a critical role in this paper. We argue that as long as we do not interrogate students’ contextual peculiarities, the road to decolonisation will continue to wind and remain slippery and, as a result, fail to realise significant transformation.

Yosso (2005) moves away from the deficit view of people of disadvantaged backgrounds and focuses on cultural knowledge, skills, abilities and contacts possessed by this group and argues that these are useful for them to navigate education. However, the skills are often unrecognised and unacknowledged in education and specifically in higher education (Yosso, 2005). Although that is the case, we acknowledge that something has been achieved in South Africa post-1994 to democratise education. This includes, among other interventions, widening access to previously marginalised groups and distribution/redistribution of resources. However, there is little evidence that these interventions have had an impact, thus giving rise to the student-led decolonisation protests.

We acknowledge Maringe and Moletsane’s (2015) research which extensively explored the concept of poverty and views it as a multifaceted concept. They framed poverty as a seven-dimensional: income, material, epistemological, employment, health, education, capability and ethical kinds of poverty (Maringe & Moletsane, 2015). Their argument is that governments, universities and schools “tend to provide single-track solutions to the poverty-stricken educational environments” and that this does not help in ameliorating poverty and deprivation (Maringe & Moletsane, 2015, p. 354). For them, “the effects are multidimensional and difficult to erase and need multipronged and multidimensional interventions” (Maringe & Moletsane, 2015, p. 355). We realise that this research was carried out in basic education in South Africa and that we have insufficient literature in higher education that speaks to poverty and deprivation. We also note that the seven dimensions of poverty overlap and interweave. In this paper, we use only three dimensions to understand poverty in higher education: the material, income and epistemological types of poverty. We have chosen these because they were seen as having sparked the 2015/2016 student protests. We are also aware of the absence of research that captures the student voice and the limited empirical evidence.

The next section defines the notion of crises and disruptions. The two concepts give an understanding of how we have framed the #FMF movement within the decolonisation debates. Without the effort to understand, bring together and interrogate the already existing evidence about student-led decolonisation protests and their impact on teaching and learning, we will not realise meaningful development in teaching in times of crises and disruption now and in the future.

## The notion of crisis

We generally view a crisis as a moment that leads to an unstable situation, consequently affecting the people concerned. Crises usually occur abruptly with no warning, with people caught unawares. As briefly discussed in the introductory section, we locate students’ protests within crises. Coombs (2007, pp. 2–3) defined a crisis as “the perception of an unpredictable event that threatens important expectancies of stakeholders and can seriously impact an organisation’s performance and generate negative outcomes”.

The concept of crisis in the Marxist notion of capitalism is where the term denoted a dangerous point for either labour or capital or both (Gamble, 2010). For Rikowski (2020), a crisis is a turning point where “systems face ruptures, radical changes or strengthening” (p. 12). Gamble (2010) emphasised that it is an event that may lead to unstable conditions and may affect an individual, a group or the whole society. Examples of crises that are externally driven include floods, earthquakes, nuclear disasters and pandemics (Rush, 2020). However, some crises, like student protests and staff withdrawal of labour, are internally driven (Maringe et al., 2020). However, internally driven crises seem to have the same impact as ones originating from outside. Chiramba (2021) argues that, when hit by crises, countries rethink and redefine a new kind of normality that allows transformation. The same applies to education systems, such as in the case of the COVID-19 pandemic and the student-led decolonisation protests in 2015/2016 which pushed the systems to rapidly adapt to new ways of doing things. The usual way of doing things is disrupted and organisations innovate at a much faster pace than they could normally have. During an emergency, they renew energy to develop new ideas and drive change (Hill, 2020). For example, during the student protests, university leaders realised that traditional top-down leadership styles were ineffective; as a result, they became more open, allowing individuals at various points of expertise to jointly lead. The involvement of various stakeholders and the need for a coordinated approach during crises are essential for successful resolution of the problem. Thus, we argue that crises are central to and necessary for bringing disruption and in the context of education, they cause epistemic disruptions.

## The notion of epistemic disruption

The term epistemic disruption is composed of two aspects: the first represents opportunity and the second barriers. Whilst the term itself can be viewed in a negative way as expensive and detracting from other priorities, a closer look shows that epistemic disruption is significant in directing education sectors and higher education in particular to act with urgency to find solutions to long-standing problems. For example, since the 2015/2016 student protests, there are great opportunities to critically reflect on students’ educational experiences. Given the debates that have arisen as a result of the protests, there are also opportunities for academics to embark on knowledge creation informed by their own social and indigenous settings as opposed to reproducing external interpretations. As with the COVID-19 pandemic, the student protests were a significant, visible and positive force of disruption. Like the strong trend to technology use during the COVID-19 pandemic, student-led protests about decolonisation have had a significant impact on “the social processes and relations of knowledge production” (Hallinger, 2019 p. 320). Protests in general serve to conscientise people to the challenges ingrained within organisations.

The second negative aspect of epistemic disruption is where internal or external forces alter the normal course of events to the extent that epistemological gains and access are curtailed or severely impacted. Such disruptions cause teaching and learning stoppages and, in severe cases, can lead to the closure of educational institutions. Those who suffer the most are students from marginalised communities as the solutions to these disruptions tend to be based on middle-class values which do not resonate with the preferences and needs of low-income students and those who suffer material poverty. An example is that of the recent COVID-19 pandemic with its abrupt turn to online teaching and learning which has tended to favour the richer students over the poorer ones (Maringe et al., 2020). Research on digitisation and equity has clearly shown that students from low socioeconomic backgrounds are faced with a myriad of challenges when it comes to the implementation of technology-driven teaching and learning environments (Rodriguez, 2009). The most common challenges are related to accessibility, affordability, connectivity, availability and usability of the online devices (Rodriguez, 2009). This also applies when universities are closed because of protests. Students who are economically and materially deprived have no other means of accessing knowledge especially when learning from home. The literature shows that learning gains for vulnerable students tend to be corroded in such times (Maringe et al., 2020). Reading levels, mathematical abilities and computational skills, which are foundational for learning, are lost during periods of disruption. We argue that these challenges result in epistemic injustice.

## Theoretical framework

### Decoloniality

This paper utilises decoloniality theory as its theoretical framework. One of the main aims of the decolonisation project is to achieve social justice and equity within higher education. However, we still see a system which continues to favour middle- and upper-class values at the expense of those from low socioeconomic backgrounds. Thus, the theory equips us with knowledge of fighting coloniality to achieve our objectives of decolonisation. Coloniality as used in post-colonial studies means continued domination and exploitation in various forms and across multiple sectors of society despite the call for decolonisation. The concept of coloniality is discussed in detail in the following section.

The discourse of decoloniality was introduced by Quijano (1990). It delinks from the colonial matrix of power (Mignolo & Walsh, 2018). Maldonado-Torres (2011, p. 5) provided an extended definition of decoloniality:

By decoloniality, it is meant here the dismantling of relations of power and conceptions of knowledge that foment the reproduction of racial, gender, and geo-political hierarchies that came into being or found new and more powerful forms of expression in the modern/colonial world struggles to bring into intervening existence another interpretation that bring forward, on the one hand, a silenced view of the event and, on the other, shows the limits of imperial ideology disguised as the true (total) interpretation of the events in the making of the modern world.

Maldonado-Torres (2016) describes decoloniality as a turn against coloniality. The discourse, as an antithesis of coloniality, demands freedom from colonisation. In 2015, students from the RhodesMustFall movement made a number of demands that permeated from their tiredness with coloniality and how it manifested itself in spaces of higher education. Among these demands were “the decolonisation of the curriculum, and socially just pedagogies and equity of access” (Postma, 2019, p. 7). These demands became one of the main goals of the protests. We use the theory of decoloniality as a lens to explore how students may be liberated from material and economic poverty, irrelevant knowledge and ways of disseminating knowledge that do not allow them to flourish.

## Review of related literature

### The theoretical and conceptual dimensions of decolonisation

Some concepts and theories are discussed in this paper to examine why it has been so difficult to achieve the long-envisaged transformation. Below, we begin by discussing transformation as consisting of three dimensions: the symbolic, the epistemological and the ideological. We argue that the three dimensions are equally and significantly important and true and that meaningful transformation should not omit any one of them.

#### Symbolic transformation

Like epistemological and ideological transformation, symbolic transformation whilst important should not be an end in itself (Maringe & Chiramba, 2021). Transformation in the context of decolonisation should go beyond symbolic transformation. The concept of symbolic transformation includes policies, strategies, renaming roads and lecture halls and recruiting black staff and Vice Chancellors among other actions which show the observable face of transformation (Maringe & Chiramba, 2021). Kotter (2009) argues that the reason why transformation always fails is that it only goes as far as, for example, formulating new policies yet true transformation is expected to go beyond that to implement the policies and alter the effectiveness of systems and processes.

Carini (1969) discussed elements of symbolic transformation as structural and involving processual elements, people capacitation and resource allocation without which, he argues, the physiology of transformation cannot be sustained. These are the visible signs or symbols of not only the desire and ambition to transform but also the anatomy that supports and provides the enabling resources for transformation (Carini, 1969). For Carini (1969), structural elements involve transforming organisational or systemic infrastructures. This includes the creation of institutional leadership and management formations to lead the transformational ambitions and also committees that deliberate and report on required institutional changes. Lastly, Carini (1969) argues that processual elements include the creation of systems, procedures, strategies and implementation plans.

The people capacitation element involves special training and continuous professional development of the agents of transformation in organisations and at the systemic level. The resource element includes allocation of adequate budgets and integrated accountability processes in their utilisation. Whilst many universities now have transformation as a key part of their agenda of overall strategic intent, there have not been many high-level appointments at the levels of DVC to steer the transformational ambitions of universities. Examination of universities’ senior level management structures suggests that transformation is often relegated to mere administrative roles which tend to deal with issues of the anatomy rather than the physiology of transformation.

#### Epistemology and epistemic transformation in universities

Epistemic transformation refers to the cosmologies of knowledge and how these contribute to a better understanding of people, how they think, feel, understand and utilise the knowledge to build a more equitable present and future for themselves (Maringe & Chiramba, 2021). The current status of the universities and how they operate entrench old patterns of exclusion (Heleta, 2016). Kotze (2018, p. 113) summarised what was said to characterise exclusion in the 2015/2016 student protests.

South African universities were cast as untransformed, crippled by institutionalised racism and curricula unsuitable for an African context and advancing a political project of African knowledge. Instead of facilitating social change and transformation, the claim is that South African universities continue to oppress the African through prioritising European and Western literature and Euro-centric views, and that this is evident in whose voices find expression in curricula and in the classroom. They favour European, American, and Anglo-Saxon authors and thinkers in philosophy and English classes as well as celebrating the advances in science by Westerners while glossing over African achievements.

In tandem with Kotze (2018), Heleta’s point is that the knowledge systems in South African universities have not meaningfully changed. Of importance to note is that the curriculum has barely changed and remains rooted in white and western ways of knowing (Heleta, 2016). Decolonising such a curriculum involves rethinking, reframing and reconstructing the curriculum to suit African ways of knowing in teaching, learning, research and assessment. As it is now, it presents the epistemic violence and hegemony of Eurocentrism.

We embrace Morrow’s call to move beyond physical access to epistemological access (Morrow, 2009). As opposed to physical access, which we argue has largely been achieved in South Africa, epistemological access provides mechanisms for ensuring that all students, whatever their socioeconomic background, gender, race or class, should enjoy equitable access to the cosmologies of knowledge and the benefits intended. Maringe and Chiramba (2021) view epistemological access as a triadic concept comprising cognitive access, access to relevant knowledge of the greatest worth and the opportunity to equitably reap the benefits of an education. What they note as a gap in the literature is that, whilst the cognitive and equitable benefits dimensions have been extensively explored, we know very little about the notion of access to knowledge of the greatest worth (Maringe & Chiramba, 2021). Reconceptualising the notion of knowledge in higher education should therefore include interrogating the aspect of worthwhile knowledge. In research carried out by Chiramba (2020), refugee students argued that the knowledge they acquire in South Africa is not relevant for their home countries and that therefore that kind of knowledge is not of worth to them when they consider going back to rebuild their countries of origin. Maringe and Chiramba (2021) also argue that worthwhile knowledge should involve the past, the present and the future of the people using it.

The main task of universities should therefore be to create, disseminate and transform knowledge into innovation for use by society (Altbach, 2016). For this to happen, there is a need to reconceptualise the discourse of knowledge transformation. Mbembe (2015) argues that epistemic transformation in African universities involves the re-centring of knowledge production systems to subvert the epistemic violence occasioned by the Western canon and recalibrating a new pluri-versal and inclusive knowledge system. In the quest to transform knowledge, questions to be asked are what and whose knowledge (Motala et al., 2021). De Sousa Santos (2015) argues that the answers often supplied to questions about knowledge are partial and very weak.

Knowledge is at the heart of social and economic development across the world and therefore, the need to transform the knowledge dimension to reflect the transition from a deeply divided and unequal society created by the apartheid and colonial regimes should be central to the transformational agenda in universities in South Africa (Teferra & Altbach, 2004). Knowledge is produced largely through research in our universities. Its production is determined by the new questions researchers must ask to reflect the needs of a transforming society, including the development and utilisation of new theories and models for its creation (Jansen, 2004). Knowledge dissemination happens largely through teaching, publication and conferences. The models and mechanisms for dissemination of knowledge have hardly changed but it is through crises and disruptions that we note some few changes. Western control over journals and over the criteria which evidences good teaching has become even tighter as local journals continue to exist on the peripheries of knowledge dissemination practices in higher education; and the criteria for high impact journals continue to prioritise Western and international journals rather than local ones (Akena, 2012).

#### Ideological transformation

Ideological transformation includes a broad societal perspective on a national identity and its preferred elements of a world view, including the theories it utilises to interrogate issues of development, progress and growth (Althusser, 1970; Marx & Engels, 1976). Baker (2009) and Giroux (2005) argue that all development and under-development are steeped in ideology. Modernity has been based on an ideology of the superiority of the Western canon. In South Africa, apartheid was an ideology which propelled separate and unequal development. Recent ideologies follow the international and globalised trends of imparting knowledge.

We value Althusser’s (1970) argument that we are because of ideology which we either recognise or misrecognise and, through those processes, we gain our identities both as producers and products of our consciousness. He further argues that a system, especially in the post-colonial world, which does not alter its ideological orientation can only reproduce the ideology which preceded it. The key ideological mechanism by which South Africa sought to transform itself was by becoming a democratic society as it sought to replace the repressive and oppressive authoritarian ideology of its apartheid predecessor. However, democracy is an underlying ideology of most of the free world and the values such as equality, equity and social justice by which it is sustained are not new to either the old or new worlds (Youngs, 2015). Although South Africa embraced the notion of Ubuntu as democratic practice (Sindane & Liebenberg, 2000), up to now, the idea has not firmly grounded in the consciousness of people and has tended to be a subject of academic discussion rather than a real underpinning framework that informs what we do, what and how we think, how we do things and how we evaluate the success and failure of our endeavours.

The clash of ideologies also has a huge impact on decolonisation. It seems with the coming of other contemporary discourses like the Fourth Industrial Revolution (4IR) and internationalisation, decolonisation is sidestepped, and we are likely to fail to achieve meaningful decolonisation because academics emulate discourses that originate from the West.

### Pedagogical transformation in teaching and learning

Omodan (2019) argues that pedagogical transformation in the new era should involve an “infusion of democracy and human rights” in teaching and learning. He views three approaches as central to pedagogical transformation: “teaching and learning as collaborative knowledge construction, teaching and learning as a critical reasoning process and teaching learning known as a disruptive caring pedagogy” (Omodan, 2019, p. 190). These should be practiced in a bid to fight the traditional ways of teaching which are anti-democratic. Omodan (2019) further argues that we need to advance democratic learning through the use of experiential and dialogical teaching methods. Dolmans et al. (2005) argue that universities should move beyond traditional methods of teaching which do not allow critical thinking but are examination-oriented despite an emphasis on twenty-first century skills. They advocate problem-based learning and discuss five principles as significant for promoting engagement, self-confidence and critical thinking in students.

1. Learning should be a constructive process: students should be active “creating meaning and building personal interpretations of the world based on individual experiences and interactions” (Dolmans et al., 2005, p. 32).
2. Learning should be a collaborative process: students should be given the opportunity to interact with each other in the knowledge making process.
3. Learning should be a contextual process: all learning should be situated so that it can be useful to the student.
4. All learning should be a self-directed process: students become actively involved in planning, monitoring and evaluate the whole process of teaching and learning.
5. Group work serves as a stimulus for interaction.

Makoelle (2021) bemoans the fact that democratic education remains bleak and educators in schools and universities are unaware of what constitutes democratic pedagogy; as a result, they still deploy the same methods they used prior to democracy.

We now move on to discuss three dimensions of deprivation.

### Three dimensions of deprivation

In this section, we discuss three dimensions of poverty (material, economic and epistemological) as central dimensions that help us to understand the contextual realities of higher education in South Africa. We begin by conceptualising the term ‘deprivation’.

The term ‘deprivation’ was used as a lens to examine “an array of social and economic issues, including poverty, poor housing conditions, and access to services” (Norris (2007) in Matheson et al., 2008, p. 678). Townsend (1987) argues that the term is used not only to analyse social conditions but also, in applied form, can be used in policy to allocate resources within a given society. Makomane (2011, p. 3) gives an extended definition of material deprivation as:

A denial of choices and opportunities, a violation of human dignity. It means lack of basic capacity to participate in society. It means not having enough to feed and clothe a family, not having a school or clinic to go to, not having a land on which to grow one’s food or a job to earn one’s living, not having access to credit. It means insecurity, powerlessness and exclusion of individuals, households and communities. It means susceptibility to violence, and it often implies living in marginal or fragile environments, without access to clean water or sanitation.

Hick (2014) argues that material deprivation and low income are the most well-known factors defining poverty. In education, material deprivation includes lack of access to material resources which promote epistemological and psychosocial capital which enhances students’ adjustment to new learning environments. The lack of appropriate clothing, shoes, mobile phones and other such resources may in itself act as an impediment to access and participation.

Income poverty refers to a situation of low or no income in a household. In the developing world, South Africa is categorised as a wealthy country, yet the majority of its population lives in income poverty (Chikoko, 2021). According to Chikoko and Mthembu (2021), about a quarter of South Africa’s population survives on government social grants, an indicator of severe income poverty. During crises and disruptions, many students and especially those from formerly marginalised backgrounds could not purchase technology to utilise during remote teaching and learning. They struggled to get the basics for survival (Chiramba, 2021).

Epistemological poverty means “being excluded, either intentionally or otherwise, from life empowering processes and those processes which increase human dignity, knowledge and understanding” (Maringe & Moletsane, 2015, p. 354).

### Poverty and higher education in South Africa

#### What are the effects of poverty and deprivation on academic achievement?

According to Letseka and Breier (2008), 50% of students who enrol in higher education in South Africa drop out before they complete their studies and that most of the students who dropped out came from low-socioeconomic backgrounds with the breadwinners earning as little as R1 600 or less a month. The study also discovered that the majority of the parents in the dropouts’ households did not have formal education, hence their epistemological poverty resulting in income poverty (Letseka & Breier, 2008). The study also found that these dropouts were not able to rely on their parents to supplement what they received from the National Student Financial Aid Scheme (NSFAS); as a result, they took part-time jobs, “adding to their stress levels and distracting them from their studies” (Letseka & Breier, 2008, p. 90). The dropout challenge followed the contours of race and privilege as more than 60% of the dropouts were black and coloured, arguing that their major reason for dropping out was lack of funding (Letseka & Breier, 2008). Failing courses came as the second major reason, which may have had two causes: a failure to engage during teaching and learning because the process was alienating for them; or the stress of income poverty and lack of time to engage with their studies, with most of them claiming to work to supplement their finances. These experiences do not align with the goals of higher education to improve the success rate.

In a recent study carried out in the Australian higher education system, McNamara et al., 2019, p. 85) argue that whilst higher education is considered a “passport out of poverty”, it is poverty that “often creates both material and cultural barriers to this access”. They also argue that childhood poverty coupled with trauma plays a significant role in how the individual will navigate higher education (McNamara et al., 2019). The decolonial discourse has been viewed as having the potential to eradicate poverty and deprivation; however, there is a strong history of resistance to post-colonial transformation, especially in South Africa. In fact, there has partial commitment to decolonise higher education despite the interventions put in place. As stated previously, we need a comprehensive decolonisation to take place in universities, with three kinds of transformation happening simultaneously: the symbolic, the epistemological and the ideological. However, for African universities, the appeal of a Eurocentric-driven educational reform which is usually better funded has always been strong. Measurement of performance is determined by the West. Continued dominance of English, unemployment and persistent poverty are enemies of decolonisation. Post-colonial countries always look up to the West.

## Conclusions and recommendations: towards a total commitment to decolonisation

We have pinpointed a number of factors that act as barriers to transformation. The first is to do with the need to reconceptualise transformation. This should be framed within three dimensions: the symbolic, the epistemological and the ideological. The literature shows that the South African higher education system has only managed to achieve the symbolic dimension and struggles to implement the other two. There has not been much transformation within the epistemological and ideological dimensions, but universities have tended to use the knowledge and ideologies we are trying to get rid of. We further argue that coloniality may be the major reason why matters continue in this way.

The second argument in the paper is to do with epistemological transformation. We developed further the points on politics of knowledge made by Jansen (2019) and viewed knowledge as consisting of physical and epistemological knowledge as well as worthwhile knowledge. Failure to unpack and understand these dimensions of knowledge leads to superficial practices within universities and an eventual failure to achieve meaningful transformation.

Thirdly, we have argued that teaching, learning and research still follow the methodologies used during colonialism and apartheid. We argued that the discourses, theories and concepts used in the post-colonial context reflect those of colonial and apartheid education systems; as a result, social justice and equity of access remain elusive in the new era. In education, we must never lose sight of the need to transform knowledge production systems to create value for the previously disadvantaged.

Lastly, we argued that something is seriously missing in the debate about transformation of higher education and that this has a huge impact on academic achievement. Poverty and its dimensions of material, income and epistemological deprivation are discussed in this paper as aspects of transformation that are little debate but have a very large effect on academic achievement. The three dimensions have been implicated in the recent decolonisation protests as seriously affecting on how students from low socioeconomic backgrounds navigate higher education.

We argue that interrogating the points discussed above may help with moving the decolonisation agenda. The starting point should be to engage in empirical research and explore further the theory of transformation as conceptualised in this paper. Secondly, academics should also aim to further unpack what we really mean by knowledge in higher education and equip themselves with the right knowledge to impart to students. Most importantly, educators should aim to understand the experiences of students from marginalised communities and accommodate them in the process of teaching and learning. The starting point should be more research into what links poverty and deprivation to educational gains. Increasing university access requires specific measures to address poverty, including financial support and pedagogies that allow students to flourish.

A pluri-versal educational focus needs to be embedded in curriculum transformation in post-colonial universities. We have to resist a chopping and replacing approach to curriculum transformation. Universities need clear strategies with budgets set aside for the decolonisation of higher education. Most importantly, we have to champion the infusion of social justice objectives into the contemporary and global issues that confront us.
